# Supplementary material for: AI-based mobile application to fight antibiotic resistance
Source: Nat Commun. 2021 Feb 19;12:1173. doi: 10.1038/s41467-021-21187-3 (PMC7895972; doi:10.1038/s41467-021-21187-3)
Supplement: Supplementary file 1 — Supplementary Information [file 41467_2021_21187_MOESM1_ESM.pdf]

AI-based mobile application to fight antibiotic resistance

Marco Pascucci, Guilhem Royer, Jakub Adamek, David Aristizabal, Laetitia Blanche, Amine Bezzarga, Guillaume Boniface-Chang, Alex Brunner, Philippe Cavalier, Christian Curel, Gabriel Dulac-Arnold, Nada Malou, Clara Nordon, Vincent Runge, Franck Samson, Ellen Sebastian, Dena Soukieh, Jean-Philippe Vert, Christophe Ambroise, Mohammed-Amin Madoui

Supplementary Note 1

The precision of automatic measurements depends on image quality. In order to obtain the best possible results on images acquired with a smartphone camera, we designed a simple protocol and acquisition setup. First of all, the environmental illumination should be homogeneous and strong enough to comfortably read a printed text (e.g. the pictures should not be taken in a dark room nor close to a window or table lamp). The Petri dish should be placed uncovered on a flat, black, non-reflecting surface (e.g. a piece of black felt). A dark background enhances the bacteria/inhibition contrast and is a standard recommendation in the EUCAST guidelines for antibiogram reading[1]. Place on top of the Petri dish a sheet of black cardboard held by two objects of equal height. A hole the size of the phone camera is pierced in the middle of the cardboard and the pictures are taken through the hole with the phone lying on the cardboard. The distance between the Petri dish and the smartphone (i.e. the stand height) is determined by the camera's field of view. It should be adjusted so that the Petri dish is well-fitted in the frame displayed by the camera.

The optics of smartphone cameras are not conceived for quantitative measurements, therefore small optical distortions are tolerated in production. However, initial exploration with standard calibration patters captured with several Android phones (Samsung A10, Huawei Y6, Pixel 3) suggested that optical distortion does not significantly impact measured diameters. In order for a smartphone camera to be suited for the AST measurements, the distortion it introduces should not impact the measurement more than the test sensibility (i.e. 1mm). In order to validate the smartphone camera, we generated and printed a fake antibiogram (see Supplementary Figure 1). The ratio between the antibiotic disk and inhibition diameters in this picture is 25/6, so the App should measure a constant diameter of 25mm for each pellet. This simple test allows a rapid validation of smartphones cameras before using them for AST analysis.

Supplementary Note 2

If the antibiotic disks are placed on the antibiogram with a dispenser, their relative position is known in advance and can be exploited[2]. Unfortunately, dispensers are not always available and

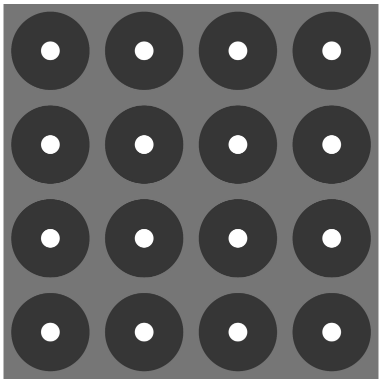

Supplementary Figure 1: Numerically generated AST picture which can be and used to check if a smartphone camera is suited for analyzing antibiograms. The diameter measured by the app should be $25\,\mathrm{mm}$ for all disks if the antibiotic disk diameter is set to $6\,\mathrm{mm}$.

placing the antibiotic disks by hand remains a common practice in many laboratories, especially in resource-limited settings. Therefore, we decided not to rely on any assumptions regarding pellet position.

The detection procedure is based on the hypothesis that the pellets are white disks: their shape is approximately circular, and the intensity levels of the associated pixels in the digitized image are high. The blue channel is taken from the cropped image of the Petri dish: we observed that in pellets usually have higher contrast from the rest of the image in the blue channel. Contrast is enhanced by histogram normalization, then an intensity threshold is applied to distinguish pellets from non-pellet pixels. The threshold is 0.97 times the maximum allowed intensity level (white).

The image is de-noised with standard mathematical morphology operations. Then, the contours of the connected components are extracted [3] and filtered according to their relative size in the image and to their thinness t:

$$\mathrm{t} = \frac{l^2}{4\pi A} \tag{1}$$

where l is the length of the contour and A is the enclosed area. By construction, $\mathrm{t} \simeq 1$ for a contour close in shape to a circle.

This eventually allows to discard bright objects such as light speckles. The contours are considered to belong to antibiotic disks if their diameter is within 1/30 and 1/4 of the image largest size and $\mathrm{th} < 1.05$.

If an antibiotic pellet is not detected automatically, the user needs to add it manually in order to continue the analysis. The detection precision influences the further analysis results. For this task we implemented a semi-automatic procedure that takes as input the approximate coordinates of the pellet center (e.g. the user clicks on the pellet) and then accurately finds the pellet center and diameter with a Hough circle transform [4]. The previously collected information of average pellet

layer type	kernel size	output size	activation
2D convolution	8	32	relu
2D convolution	4	64	relu
2D convolution	2	64	relu

Supplementary Table 1: Convolution layers in the CNN model used to classify the antibiotic disk labels

size in the image is used to filter in the transform space.

Supplementary Note 3

Antibiotic name recognition is treated as a multi-class problem because there are as many codes to be recognized as there are antibiotics. This supervised learning problem is tackled via a convolutional neural network (CNN) trained using 18000 images of antibiotic disks captured with a smartphone camera. More precisely, the images were split in two sets: a training set of 14000 images and a test set of 4000 images used also for validation.

The set contains 65 different labels corresponding to the pellets of two manufacturers (Liofilchem and i2a). We estimate that across the major disks manufacturers, there are over $\simeq 200$ different labels. Our model should eventually be able to identify all of those labels.

The input images are first converted to gray-scale and resized to 64x64 pixels. Then, the intensity value of each pixel is normalized by subtracting the mean intensity and dividing by the intensity standard deviation. The model takes a standardized pellet image as input and returns an array of 65 values between 0 and 1 normalized to 1. These values are interpreted as the probability of belonging to a specific class (i.e. label for a given antibiotic).

The model is a CNN with three convolution layers described in Supplementary Table 1. Each convolution layer is followed by max-pooling with a down-scaling factor of 2. The classification output is obtained with a random 50% dropout and a final dense layer of 65 units. We used cross-entropy as loss function.

The model is evaluated primarily on its accuracy. Since the data set doesn't cover all the antibiotic codes that could be encountered in a real use case, a second metric was introduced to evaluate the model behavior on unknown labels (hereafter "outliers"). To simulate this behavior, 5 classes (each class corresponds to a label) are held out from the dataset and the model is trained on the remaining 60. After defining a threshold on the model output, we evaluate:

- for the 60 classes from the test data
  - the accuracy on the predictions above the threshold (accuracy on known labels)

    - the percentage of samples that fell below the threshold (false negative rate on known labels)

- for the 5 classes held out and taken from the test data

    - the percentage of samples that fell above the threshold (false positive rate on outliers)

A single model showed good accuracy, but a simple threshold on the output values was not good enough to minimize classification errors of outliers. Therefore, we developed an ensemble model with an entropy threshold to achieve a better trade-off between accuracy and outlier classification error. We trained ten identical instances of this model with same hyper-parameters, same training data, but different random initialization. The models were trained using Tensorflow[5] in Python.

Classification is obtained by feeding the same pre-processed antibiotic disk image to each model of the ensemble, the ten classification outputs are averaged, and the information entropy of the average is calculated. If the entropy is larger than a fixed threshold, the $argmax$ of the output average determines which classes the label belongs to. Otherwise, the label is considered as unknown. The threshold is chosen to optimize the trade-off between optimizing accuracy and minimizing out-of-distribution error. From the user point of view, if a disk is classified as unknown, the users must provide the label based on visual inspection. The list of possible labels are sorted according to their output prediction value, which usually brings to the top the best candidates in case of false negatives (known pellets classified as unknown).

The ensemble model achieves 99.97% accuracy on the test set. Using an ensemble of 10 models and an entropy threshold, we achieved: 100% in-distribution accuracy, 5% in-distribution false negative rate and 1% out-of-distribution false positive rate.

Additionally, the pictures of antibiogram set A1 can also be considered as a true test set because the model never saw them during training. The model reaches 100% accuracy on this test set.

Supplementary Note 4

Diameter measurement consists of three steps: image pre-processing, intensity radial profile extraction and segmentation.

Pre-processing

The growth medium of the Petri dish is automatically determined based on the saturation, hue and intensity of the Petri dish image. First, we calculate the mean saturation, hue and intensity

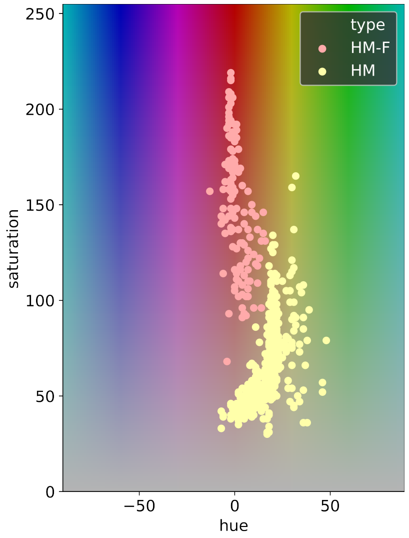

Supplementary Figure 2: distribution of the mean HUE and saturation values of the AST pictures in data sets A1 and A2. Each point represents one image. Dataset A2 contains only pictures of blood-enriched culture medium AST, which is red, in contrast to standatd MH culture medium which appears white-yellow.

over the $50\,\%$ most saturated pixels in the image, then we use these values to classify the growth medium. The classifier is built with a logistic regression on more than 600 labeled images (see Supplementary Figure 2) and achieves $100\,\%$ accuracy on this data set. If a red growth medium (HM-F) is detected, we use only the blue and green channels of the image when converting it to gray scale, in order to enhance the output contrast. Otherwise all three channels are used.

Successively the image is pre-processed for the diameter measurement. The Petri dish image is down-scaled to a fixed resolution of $10\,\mathrm{pixel/mm}$, which provides a reasonable trade off between precision and measurement speed. Then, all the pixels belonging to the plate borders are masked. This eliminates disturbing light reflections (due to surface tension, the agar is not flat close to the plate's borders). The plate image is segmented in Regions Of Interest (ROIs), rectangular sub-images centered on each antibiotic disk. Each ROI includes the area surrounding the disk up to the first-neighboring disks.

For the whole image first and then for each region of interest, a k-means classification of the pixel intensity is performed with k=2 (inhibition and bacteria) and the center values are stored. Since the k-means classification with k=2 is not optimal for very large inhibition zones or zones with no inhibition at all, in this case the k-means centers calculated on the whole image are used.

Radial profiles

The intensity of the pixels around the antibiotic disk is observed for each ROI in order to calculate a radial intensity profile $I(r)$. The profile extraction procedure takes into account both the intensity

and the amount of bacteria at a given r. Without considering any privileged direction, all pixels at a given distance r from the antibiotic disk center are considered. Then $I(r)$ is given a score between 0 and 1 depending on the number n_b of bacteria pixels. Bacteria pixels are defined as those pixels which have an intensity above the threshold I_{th}. The bacteria intensity threshold is calculated as:

$$I_{th} = k_0 + (k_1 - k_0)(1 - s), \quad (2)$$

where k_0 and k_1 are the local k-means centers for inhibition and bacteria pixel intensities. s is defined as the reading sensibility. It can be fixed between 0 and 1 or automatically determined based on the image local and global contrast c_l. Contrast is defined as ($c = k_1 - k_0$), local contrast is based on local k-means (over the antibiotic disk ROI) and global contrast on global k-means (whole image).

If automatically determined, s is equal to 0.05 for very low contrast images (contrast$\leq$25) where the signal/noise ratio is low, otherwise

$$s = \frac{1}{2}\left[\left(\frac{c_g - c_l}{c_g}\right)^3 + 1\right]. \quad (3)$$

If the local and global contrast values are similar $s \simeq 1/2$. Otherwise, it is adapted according to the ratio c_l/c_g in order to compensate noise and local intensity variability in the image.

The critical number of bacteria pixels n_b represents a spatial threshold that allows detecting the presence of bacteria at distance r even if the shape of the inhibition zone is not circular (e.g. inhibition overlap and plate borders). We choose n_b equivalent to $1\,\mathrm{mm}$ in the image scale, which corresponds to the diameter precision requested by the test.

Finally, $I(r)$ is segmented using a least-square fit of a step function with one degree of freedom: the change-point, which is interpreted as the inhibition zone radius in pixels. A maximum inhibition diameter can be specified by the user, in this case the reported diameters would be capped by that maximum (40mm by default).

The absolute diameter difference between automatic measurements and control on the test antibiotics sets are reported in Supplementary Table 2. The larger absolute diameter difference in A1 and A2 can be attributed to the fact that the control diameter (measured by a commercial automatic system) was not systematically adjusted if adjustment did not have an effect on the categorization.

Published algorithms for measuring diameters

Among the different algorithms for this specific task we could find in the literature[6,11] do not prove to be resilient to the common problems of non-homogeneous or non-circular inhibition zones.

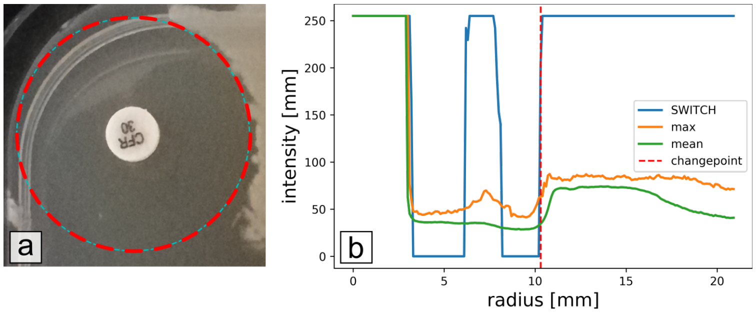

Supplementary Figure 3: Radial intensity profile extraction. The region of interest of an antibiotic pellet is displayed in (a). Different radial profiles are plotted in (b) by taking the max or mean intensity value at distance r. The blue profile is calculated by the SWITCH algorithm presented in this paper. The peak of the signal in the inhibition zone is due to light reflection on the agar, but the segmentation correctly ignores it. In the mean profile, since the borders of the inhibition zone are not flat, the signal ramps up smoothly, introducing uncertainty in the change-point location, then the signal drops because of inhibition zone overlap. These problems do not affect SWITCH.

AST set	control	images	antibiotics	a.d.d. quantiles [mm]		
				25%	50%	75%
A1						
overall	SIRscan	572	8168	0.8	1.8	3.0
standard	SIRscan	561	8042	0.8	1.8	3.0
problematic	SIRscan	9	126	2.4	6.3	20.8
A2						
overall	SIRscan	75	649	0.6	1.4	2.8
standard	SIRscan	73	620	0.6	1.4	2.6
problematic	SIRscan	2	29	11.0	17.0	22.0
A3						
overall	manual	64	784	0.0	0.8	1.4
overall	manual av.	64	98	0.1	0.6	1.3

Supplementary Table 2: Absolute diameter difference (a.a.d.) between automatic measurement and control.

system	licence	control	readings	agreement (%)	disagreement (%) very major	major	minor
the App	free	SIRscan (A1-2)	7868	90.0	0.7	5.8	3.5
the App	free	manual (A3)	776	95.0	0.4	0.9	3.5
the App	free	manual av. (A3)	97	98.0	1.0	0	1.0
RPAA[6]	free	manual	2552	95.5	0.3	0.4	2.7
AIA[2]	free	manual	756	88.0	1.0	2.0	3.0
AntibiogramJ[7]	free	manual	720	87.0	2.0	5.0	6.0
Aura[8]	commercial	manual	4098	82.4	4.9	16.0	4.9
Osiris[9]	commercial	manual	1592	91.6	3.8	0.9	3.0
Adagio[10]	commercial	manual	1548	94.8	0.8	3.3	1.4

Supplementary Table 3: Comparison of categorization agreement/disagreement of the App's performance shown in this paper versus similar studies concerning other automatic systems. This comparison should be taken with caution because the datasets used in these studies are different under many aspects (e.g. species distribution, manual measurement procedure, image acquisition and outliers dropout).

			a.d.d. quantiles [mm]			disagreement		
resolution [megapixel]	missed atb disks	unread labels	25%	50%	75%	very major	major	minor
original (12.8)	0	0	0.12	0.60	1.27	1	0	1
9.0	0	0	0.13	0.55	1.25	1	0	1
6.0	0	1	0.15	0.53	1.25	1	0	1
3.0	0	0	0.15	0.57	1.19	1	0	1
1.0	6	2	0.31	0.53	1.18	1	0	1

Supplementary Table 4: Effect of resolution degradation on dataset A3 (averaged). The results are substantially the same up to resolution = 3 Megapixel. At resolution = 1 megapixel, 6 antibiotic disks are missed out of the total 98, but the classification error remains constant.

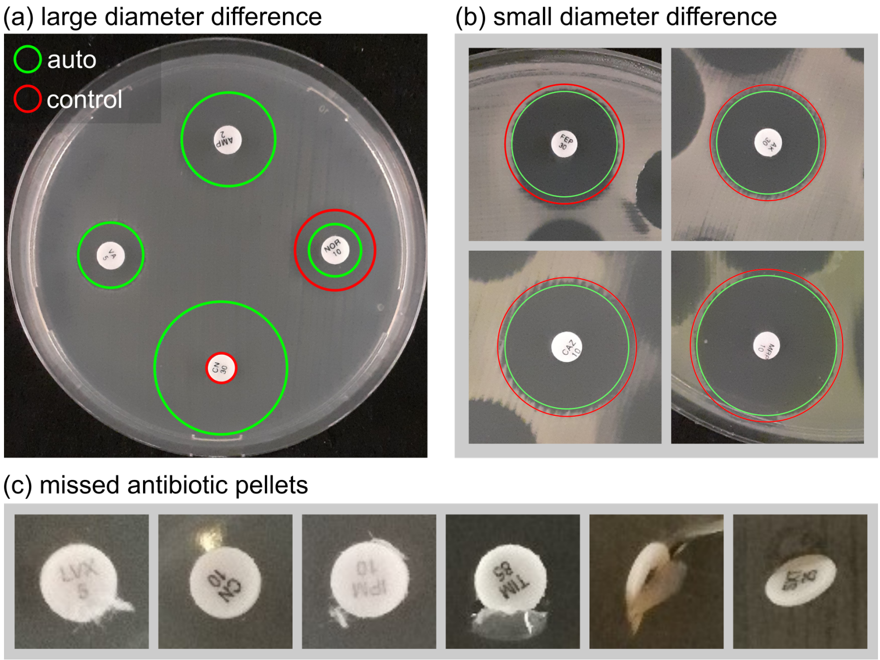

Supplementary Figure 4: Automatic reading mistakes. Cases of large mistakes in inhibition diameters happen sometimes when the bacteria-inhibition contrast is low (a). Smaller diameter differences (b) between the App and reference are probably due to the intrinsic measurement method: by visualizing a circle the App increases the measurement precision. Figure (c) shows some examples of pellets that where misclassified. The three last pellets have broken the agar, antibiotic diffusion is not assured to be uniform hence they should be discarded according to the AST reading guidelines.

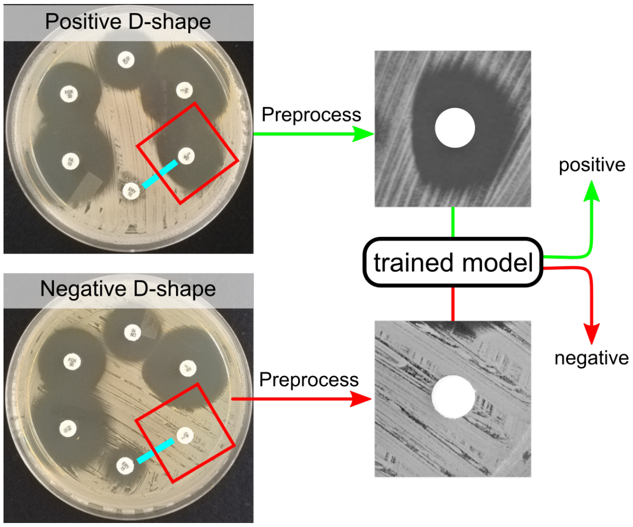

Supplementary Figure 5: D-Zone prediction workflow. The inhibition zone of the antibiotic which may signal a resistance mechanism is cropped out of the picture. This sub-image is preprocessed (rotated and gray-scaled) and presented to a trained model for classification.

Same for [12], which takes into account both intensity and texture with a Student's t-test, but is sensitive to noise and assumes that the inhibition and bacteria have homogeneous textures. A recent work[2] calculates a threshold for segmentation only along 4 segments centered on the antibiotic pellet, with the risk of losing information.

Supplementary Note 5

The raw input to the training pipeline consists of photos of entire Petri dishes used for AST. Photos were taken using a smartphone camera at an MSF field hospital in Amman, Jordan during the course of regular AST processing. As a result, the photos represent similar conditions to those under which the App will eventually be used. Only relevant examples (those containing adjacent pairs of Clindamycin and Erythromycin pellets) are considered. The training data includes 69 positive and 143 negative examples, each image is labeled as “D-Zone or “not D-Zone.

First, the photos are preprocessed. The first four preprocessing steps aim to standardize input characteristics across examples (see Supplementary Figure 5):

1. Identify the Clindamycin and Erythromycin pellets using the pellet label recognition strategy described above.

2. Rotate the image such that Erythromycin and Clindamycin are aligned along the x-axis, with Erythromycin on the left.

3. Crop out the biggest region surrounding Clindamycin that does not include other pellets.
4. Overlay the Clindamycin pellet with a $6\,\mathrm{mm}$ white circle in order to mask the printed text, which has no interest in the classification problem. In the interest of a small, lightweight model, the final two steps simply reduce input size without loss of model accuracy. As a result, the numerical input to the machine learning model is simply a 32x32 integer matrix, where each entry is a value between 0 (black) and 255 (white).
5. Convert the image from color to gray-scale
6. Downsize the image to $32\,\mathrm{pixel} \times 32\,\mathrm{pixel}$.

We report the model's train and test accuracy, i.e. the proportion of examples for which the model correctly predicts the known label. Since the data-set is small, a 70/30 split results in 148 training samples and 64 test samples, therefore we evaluate model structures by retraining the same model structure on multiple different train/test splits. However, the model is never tested on the same images as it was trained. This approach allows us to compare different model architectures, whereas a single train/test split would result in ties.

Models were trained using TensorFlow in Python. After experimenting with multiple neural network architectures, the architecture with the best performance has the following characteristics:

- Input size of $32\,\mathrm{pixel} \times 32\,\mathrm{pixel}$,
- 73,309 total trainable parameters,
- Binary cross-entropy loss function
- Adam optimization,
- input size of $32\,\mathrm{pixel} \times 32\,\mathrm{pixel}$,
- layers: 3x 2-D convolution layers rectified linear unit activation, with 32, 64, and 128-dimensional outputs respectively,
- Dropout layer to avoid over-fitting,
- Densely-connected layer of size 50 with sigmoid activation (for binary classification).

Across 30 randomly-chosen train/test splits, the best model achieves on average 100% training accuracy and 99.74% test accuracy. Other metrics give similar results (F1 99.65% and AUC 1.0), as expected given the high accuracy score.

D-Zone detection is a relatively easy ML problem, obtaining 99.74% test accuracy with only 212 training images. The D-Zone model has not been evaluated on out-of-distribution images.

Data-set name	D1	D2
acquisition device	smartphone camera	SIRscan system
Number of ESBL positive examples	241	1344
Number of ESBL negative examples	181	818
Center pellet	Amoxicillin/Clavulanic acid 30 µg	Amoxicillin/Clavulanic acid 30 µg or Ticarillin/Clavulanic acid 75+10 µg

Supplementary Table 5: Training data-sets for the synergy model.

Supplementary Note 6

For the ESBL problem, we have access to two distinct data-sets (see Supplementary Table 5. Each data-set consists of images of AST plates. Each image contains pellets arranged to perform a Double Disk Synergy test and each is labeled as "ESBL" or "not ESBL". The two data-sets differ highly in different aspects such as image quality and intensity contrast, bacteria culture texture, pellet arrangement, specific antibiotics used.

Before training a neural network on the images, we preprocess them to standardize across images and extract relevant regions:

1. Crop out a 35mm region surrounding amoxicillin-clavulanate. In both our data-sets, 35mm is sufficiently large to encompass amoxicillin-clavulanate and the surrounding 3rd-generation cephalosporins.

2. Convert the image to gray scale.

3. Normalize image contrast such that the intensity threshold is similar across all images.

4. Overlay the amoxicillin-clavulanate pellet with a pure black circle.

Alternative preprocessing approaches did not introduce significant improvement of the prediction accuracy. For example we tested: 1. blur the image to eliminate unnecessary details such as bacteria streaks or 2. classify each pixel as bacteria vs. inhibition using an intensity threshold computed by k-means clustering (we will refer to this strategy as heavy preprocessing in this section).

As in the case of D-Zone, train and test accuracy is reported. However, since in the ESBL case we have access to two distinct data-sets, we also examine model transferability. Therefore, we examine train and test accuracy within each of the following setups:

train set (%)		test set (%)		preprocessing	accuracy (%)		F1	AUC
D1	D2	D1	D2		train	test		
70	-	30	-	light	99.83	98.04	98.89	99.84
-	70	-	30	light	98.89	97.98	99.06	99.70
100	-	-	100	light	99.93	66.77	76.90	67.17
-	100	100	-	light	99.75	69.23	62.09	64.75
100	-	-	100	heavy	99.97	58.96	NA	NA
-	100	100	-	heavy	99.59	59.16	NA	NA

Supplementary Table 6: ESBL model accuracy results.

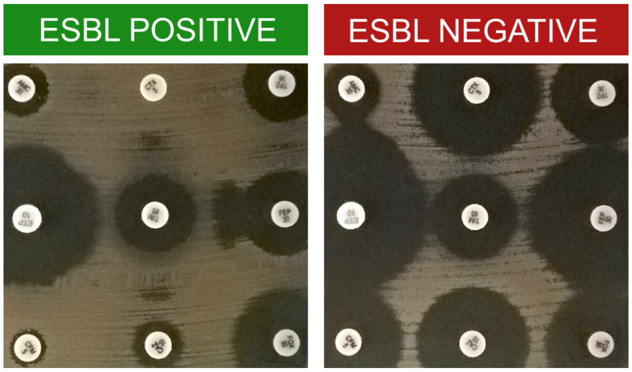

Supplementary Figure 6: Examples of ESBL positive and negative cultures.

- Train and test on disjoint sets of images drawn from the same data-set
- Train and test on disjoint sets of images, where both the test and train sets are drawn from the combination of both data-sets
- Train on one data-set and test on the other

The model is over 97% accurate when its test set is drawn from the same distribution as its training set. However, when the training and test sets are of different origin, the model performs no better than random. Out-of-distribution examples are of interest because we cannot control the quality of images that the app will eventually be used to analyze. Specifically to address this problem, we tested the heavy preprocessing approach but did not improve cross-data-set accuracy. As more training examples are added, model accuracy increases only until 300 training examples are used. Additionally, augmenting the training data-set by transforming (e.g., rotating) images did not improve validation accuracy. Therefore, obtaining more similar data is not expected to improve results. The results obtained with other metrics (F1 and ROC AUC) justified by the unbalanced data classes, confirm good performance for intra-dataset models and a much lower performance for inter-dataset models.

ESBL classification is a much more difficult machine learning problem than D-Zone classification (see Supplementary). This difficulty might be due to the very large variability in the ESBL-positive examples (see Supplementary Figure 6). While D-Zone examples are all very similar to each other, ESBL-positive examples may show different shapes, depending on the relative position and distance of the involved antibiotic disks, on the texture of the bacteria and on the quality of the picture.

References

1. EUCAST. Eucast disk diffusion method. European Committee on Antimicrobial Susceptibility Testing (EUCAST) (2020). https://eucast.org/ast_of_bacteria/disk_diffusion_methodology/.

2. Costa, L. F. *et al.* Development of an automatic identification algorithm for antibiogram analysis. *Computers in Biology and Medicine* **67**, 104–115 (2015).

3. Suzuki, S. & be, K. Topological structural analysis of digitized binary images by border following. *Computer Vision, Graphics, and Image Processing* **30**, 32–46 (1985).

4. Duda, R. O. & Hart, P. E. Use of the hough transformation to detect lines and curves in pictures. *Commun. ACM* **15**, 11–15 (1972).

5. Abadi, M. *et al.* TensorFlow: Large-scale machine learning on heterogeneous systems (2015). Software available from tensorflow.org.

6. Hejblum, G., Jarlier, V., Grosset, J. & Aurengo, A. Automated interpretation of disk diffusion antibiotic susceptibility tests with the radial profile analysis algorithm. *Journal of Clinical Microbiology* **31**, 2396–2401 (1993). https://jcm.asm.org/content/31/9/2396.full.pdf.

7. Alonso, C. *et al.* Antibiogramj: A tool for analysing images from disk diffusion tests. *Computer Methods and Programs in Biomedicine* **143**, 159–169 (2017).

8. Lestari, E. S. *et al.* Comparison of the accuracy of disk diffusion zone diameters obtained by manual zone measurements to that by automated zone measurements to determine antimicrobial susceptibility. *Journal of Microbiological Methods* **75**, 177–181 (2008).

9. Sánchez, M., del Saz, B. S., Loza, E., Baquero, F. & Cantón, R. Evaluation of the osiris video reader system for disk diffusion susceptibility test reading. *Clinical Microbiology and Infection* **7**, 352–357 (2001).

10. Idelevich, E. A. *et al.* Evaluation of an automated system for reading and interpreting disk diffusion antimicrobial susceptibility testing of fastidious bacteria. *PLOS ONE* **11**, 1–8 (2016).

11. Salgado, L., Menendez, J. M., Rendon, E., Garcia, N. & Ruiz, V. Automatic antibiograms inhibition halo determination through texture and directional filtering analysis. In *Proceedings 2001 International Conference on Image Processing (Cat. No.01CH37205)*, vol. 2, 629–632 vol.2 (2001).

12. Gavoille, A., Bardy, B. & Andremont, A. Measurement of inhibition zone diameter in disk susceptibility tests by computerized image analysis. *Computers in Biology and Medicine* **24**, 179–188 (1994).
