# Supplementary material for: AI-based mobile application to fight antibiotic resistance
Source: Nat Commun. 2021 Feb 19;12:1173. doi: 10.1038/s41467-021-21187-3 (PMC7895972; doi:10.1038/s41467-021-21187-3)
Supplement: Supplementary file 3 — Description of Additional Supplementary Files [file 41467_2021_21187_MOESM3_ESM.pdf]

Tile: Supplementary Movie 1

Description: In this video, Dr. Royer (microbiologist) uses a Smartphone to analyze disk diffusion Antibiotic Susceptibility Test from a Petri dish. He assembles a very simple cardboard setup to take the picture of a Petri dish and illustrates the analysis process from disk measurement to susceptibility interpretation.
