# Supplementary material for: AI-based mobile application to fight antibiotic resistance
Source: Nat Commun. 2021 Feb 19;12:1173. doi: 10.1038/s41467-021-21187-3 (PMC7895972; doi:10.1038/s41467-021-21187-3)
Supplement: Supplementary file 5 — Reporting Summary [file 41467_2021_21187_MOESM5_ESM.pdf]

nature research

Corresponding author(s): Marco Pascucci

Last updated by author(s): Jan 6, 2021

Reporting Summary

Nature Research wishes to improve the reproducibility of the work that we publish. This form provides structure for consistency and transparency in reporting. For further information on Nature Research policies, see our Editorial Policies and the Editorial Policy Checklist.

Statistics

For all statistical analyses, confirm that the following items are present in the figure legend, table legend, main text, or Methods section.

n/a	Confirmed	
[ ]	[x]	The exact sample size (*n*) for each experimental group/condition, given as a discrete number and unit of measurement
[ ]	[x]	A statement on whether measurements were taken from distinct samples or whether the same sample was measured repeatedly
[x]	[ ]	The statistical test(s) used AND whether they are one- or two-sided<br>*Only common tests should be described solely by name; describe more complex techniques in the Methods section.*
[x]	[ ]	A description of all covariates tested
[x]	[ ]	A description of any assumptions or corrections, such as tests of normality and adjustment for multiple comparisons
[ ]	[x]	A full description of the statistical parameters including central tendency (e.g. means) or other basic estimates (e.g. regression coefficient) AND variation (e.g. standard deviation) or associated estimates of uncertainty (e.g. confidence intervals)
[x]	[ ]	For null hypothesis testing, the test statistic (e.g. *F*, *t*, *r*) with confidence intervals, effect sizes, degrees of freedom and *P* value noted<br>*Give P values as exact values whenever suitable.*
[x]	[ ]	For Bayesian analysis, information on the choice of priors and Markov chain Monte Carlo settings
[x]	[ ]	For hierarchical and complex designs, identification of the appropriate level for tests and full reporting of outcomes
[x]	[ ]	Estimates of effect sizes (e.g. Cohen's *d*, Pearson's *r*), indicating how they were calculated

Our web collection on statistics for biologists contains articles on many of the points above.

Software and code

Policy information about availability of computer code

Data collection	Software was used only for the collection of AST images.<br>Images in data-sets A1 and A2 were collected with the Android Camera application v.10.<br>Images in data-set A3 where collected with the application presented in the paper (v0.17)
Data analysis	The custom AST image processing library v1.0 was used. The Library uses OpenCV v4.0.1 and TensorFlow v1.14.<br>The image processing library (C++ and Python) described in this paper is distributed as open-source software, available at https://github.com/mpascucci/AST-image-processing. Version 1.4 of the library was used for the data analysis reported in the paper.

For manuscripts utilizing custom algorithms or software that are central to the research but not yet described in published literature, software must be made available to editors and reviewers. We strongly encourage code deposition in a community repository (e.g. GitHub). See the Nature Research guidelines for submitting code & software for further information.

Data

Policy information about availability of data

All manuscripts must include a data availability statement. This statement should provide the following information, where applicable:

- Accession codes, unique identifiers, or web links for publicly available datasets
- A list of figures that have associated raw data
- A description of any restrictions on data availability

The datasets analysed during the current study are available at this URL: \url{http://stat.genopole.cnrs.fr/ast.zip}. The data used for training the ESBL and D-SHAPE proof-of-principle models are available from The MSF Foundation upon reasonable request.

Field-specific reporting

Please select the one below that is the best fit for your research. If you are not sure, read the appropriate sections before making your selection.

- [x] Life sciences
- [ ] Behavioural & social sciences
- [ ] Ecological, evolutionary & environmental sciences

For a reference copy of the document with all sections, see nature.com/documents/nr-reporting-summary-flat.pdf

Life sciences study design

All studies must disclose on these points even when the disclosure is negative.

Sample size	Sample size, i.e. the number of analyzed AST plates, was chosen to be comparable with other studies of the same kind published in literature.
Data exclusions	No data were excluded from the analysis
Replication	The image analysis is completely deterministic, this grants replication.<br>We provided the results for every single image in order to check this.
Randomization	We did not define samples groups in this study.
Blinding	The technicians that measured the control diameters did not have any sort of experience with the application because we did not want to influence their control measurements. No further blinding was necessary for this study.

Reporting for specific materials, systems and methods

We require information from authors about some types of materials, experimental systems and methods used in many studies. Here, indicate whether each material, system or method listed is relevant to your study. If you are not sure if a list item applies to your research, read the appropriate section before selecting a response.

Materials & experimental systems

n/a	Involved in the study	
x		Antibodies
x		Eukaryotic cell lines
x		Palaeontology and archaeology
x		Animals and other organisms
x		Human research participants
x		Clinical data
x		Dual use research of concern

Methods

n/a	Involved in the study	
x		ChIP-seq
x		Flow cytometry
x		MRI-based neuroimaging
